# Expression of the Domain Cassette 8 *Plasmodium falciparum* Erythrocyte Membrane Protein 1 Is Associated with Cerebral Malaria in Benin

**DOI:** 10.1371/journal.pone.0068368

**Published:** 2013-07-29

**Authors:** Gwladys I. Bertin, Thomas Lavstsen, François Guillonneau, Justin Doritchamou, Christian W. Wang, Jakob S. Jespersen, Sem Ezimegnon, Nadine Fievet, Maroufou J. Alao, Francis Lalya, Achille Massougbodji, Nicaise Tuikue Ndam, Thor G. Theander, Philippe Deloron

**Affiliations:** 1 Institut de Recherche pour le Développement (IRD), UMR216-Mère et enfant face aux infections tropicales, Paris, France; 2 PRES Paris Sorbonne Cité, Université Paris Descartes, Paris, France; 3 Centre for Medical Parasitology at Department of International Health, Immunology and Microbiology, University of Copenhagen and at Department of Infectious Diseases, Copenhagen University Hospital (Rigshospitalet), Copenhagen, Denmark; 4 3P5 proteomics facility, Université Paris Descartes, Paris, France; 5 Centre d’Étude et de Recherche sur le Paludisme Associé à la Grossesse et l′Enfance (CERPAGE), Cotonou, Bénin; 6 Paediatric Department, Mother and child hospital (HOMEL), Cotonou, Bénin; 7 Paediatric Department, Centre National Hospitalo-Universitaire (CNHU), Cotonou, Bénin; Université Pierre et Marie Curie, France

## Abstract

**Background:**

*Plasmodium falciparum* erythrocyte membrane protein-1 (*Pf*EMP-1) is a highly polymorphic adherence receptor expressed on the surface of infected erythrocytes. Based on sequence homology *Pf*EMP-1 variants have been grouped into three major groups A-C, the highly conserved VAR2CSA variants, and semi-conserved types defined by tandem runs of specific domains (“domain cassettes” (DC)). The *Pf*EMP-1 type expressed determines the adherence phenotype, and is associated with clinical outcome of infection.

**Methods:**

Parasite isolates from Beninese children or women presenting with, respectively, CM or PAM were collected along with samples from patients with uncomplicated malaria (UM). We assessed the transcript level of *var* genes by RT-qPCR and the expression of PfEMP-1 proteins by LC-MS/MS.

**Results:**

*Var* genes encoding DC8 and Group A *Pf*EMP-1 were transcribed more often and at higher levels in cerebral malaria *vs.* uncomplicated malaria patients. LC-MS/MS identified peptides from group A, DC8 *Pf*EMP-1 more frequently in cerebral malaria than in uncomplicated malaria and pregnancy-associated malaria samples.

**Conclusion:**

This is the first study to show association between *Pf*EMP-1 subtype and disease outcome by direct analysis of parasites proteome. The results corroborate that group A and specifically the *Pf*EMP-1 types DC8 are universally associated with cerebral malaria. This is a crucial observation for promoting studies on malaria pathogenesis.

## Introduction

Virulence of *Plasmodium falciparum* is linked to the sequestration of parasites in the host’s deep organ vasculature caused by cytoadherence of parasite infected erythrocytes (iE) to endothelial cell receptors [1], [2]. This tissue tropism is mediated by members of the *Plasmodium falciparum* erythrocyte membrane protein 1 (*Pf*EMP-1) family expressed at the iE surface [3], [4]. *Pf*EMP-1 molecules have diverged to bind different endothelial receptors, including ICAM-1, VCAM, CD36, and the glycosaminoglycan Chondroitin-Sulfate A (CSA) [5], [6]. Binding specificity of *Pf*EMP-1 is thought to be associated with clinical syndromes. iE binding to CSA is specifically associated with pregnancy-associated malaria (PAM) [7], and adhesion to ICAM-1 appears to be associated with cerebral malaria (CM) [8], [9].

The *var* genes, coding for *Pf*EMP-1 proteins, are divided into three main groups A-C, the conserved VAR2CSA variants and the intermediate groups B/A and B/C defined by their conserved 5′ upstream sequences (UPS) and complexity of the encoded domain structure [10], [11]. DBL “Duffy Binding-Like” and CIDR “Cysteine-Rich Inter Domain Regions” domains are classified by sequence homology into DBLα-ζ and CIDRα-δ domains, further subdivided into 147 subtypes (e.g. DBLα1.1). Domains subtypes at the N-terminal part of *Pf*EMP-1 molecules (*i.e*. DBLα-CIDRα-DBLβ/DBLγ domains) are associated with the UPS type, whereas domains in the C-terminal (i.e. DBLδ-CIDRβ/γ-DBLε-DBLζ) are not. The alignment of domain subtype architectures of 399 known *Pf*EMP-1s revealed a set of 21 conserved domain compositions called domain cassettes (DC) [12].

Infections causing severe malaria in children are also linked to the expression of a restricted subset of *Pf*EMP-1 [13]–[16]. The identification of specific *Pf*EMP-1 types involved in the sequestration during CM has been particularly challenging due to the great diversity of the *var* gene family. Nevertheless, it is well established that A and B/A *var* genes are over-expressed in parasites isolated from patients suffering from severe malaria [17]–[19], and recently transcription of *var* genes encoding DC8 and DC13 *Pf*EMP-1 was associated with parasites isolated from Tanzanian children suffering from CM [20]. DC8 is a four domain composition characterized by DBLα2- CIDRα1.1- DBLβ12- and DBLγ4/6- domains, whereas the DC13 is defined by the tandem domains DBLα1.7-CIDRα1.4. Two studies found that *P. falciparum* parasites expressing DC8 *Pf*EMP-1 had superior brain endothelial cell binding capabilities over parasites expressing other *Pf*EMP-1 [21], [22]. Although these studies associate DC8 and DC13 *Pf*EMP-1 with CM, it is important for future studies of pathogenesis and development of vaccine constructs in particular, to establish if these *Pf*EMP-1 variants precipitate severe disease in other regions. We investigated the *Pf*EMP-1 types expressed by parasites from subjects with CM, uncomplicated malaria (UM) or PAM in Benin, using both *var* gene transcript levels measurement and a proteomic approach.

## Materials and Methods

### Ethic Statement

Ethical clearance was obtained from the Institutional Ethics Committee of the Faculté des Sciences de la Santé at the Abomey-Calavi, University in Benin. All patients were included after written informed consent from themselves or their guardian. The medical team of each health facility managed patients where adequate anti-malarial treatment was administered according to the national malaria program policy.

### Study area and Patients Enrolment

Patients were enrolled at Cotonou in southern Benin over the 2011 malaria transmission season (May to mid-August). Malaria transmission in Cotonou is approaching 33 infecting bites per person per year [23]. Cerebral malaria (CM) patients and pregnant women presenting with pregnancy associated malaria (PAM) were included in the Centre national hospitalier and universitaire (CNHU) and the Mother and child hospital (Hôpital de la Mère et de l’Enfant Lagune), respectively. Uncomplicated malaria (UM) patients were enrolled in the health centre of Come, 70 km of Cotonou.

Uncomplicated malaria (UM) was defined as the combination of fever (tympanic temperature ≥37.8°C), confirmed presence of *P. falciparum* infection, and absence of any severity sign as defined by the WHO [24]. Cerebral malaria (CM) was defined by a Blantyre score at diagnosis ≤2 combined with a coma duration of six hours at least, and confirmed presence of *P. falciparum* infection with exclusion of other cause for coma, particularly meningitis. Patients and pregnant women at delivery were all screened for malaria infection by rapid diagnostic test for *P. falciparum* (Malaria Quick test, Cypress Diagnostics, Langdorp, Belgium). A questionnaire was administered to the children′ guardian and woman to collect social data, disease history, and treatment received before hospital admission.

Peripheral venous blood sample (5 ml) was collected from all study individuals in a vacutainer tube containing EDTA, and a placental tissue was obtained after delivery. Giemsa-stained thick blood film confirmed *P. falciparum* infection, and parasitaemia was quantified by counting against 200 leucocytes assuming a mean of 8,000 leucocytes per millimeter of blood.

### RNA Collection

Ring stage parasites were conserved either in Trizol (Invitrogen) stored at −80°C or as dried spots on Whatmann 3MM filter paper stored at room temperature. Total RNA from peripheral iE was prepared from Trizol samples followed by treatment with DNAse 1 (Sigma) for 15 min at 37°C. Absence of DNA in RNA samples was confirmed by stable base fluorescence after 30 cycles of real-time PCR with *seryl-tRNA synthetase* primers [25]. DNA-free RNA was reverse transcribed with random hexamer primers and Superscript II enzyme (Invitrogen) for 10 min at 25°C, 50 min at 42°C, and 15 min at 70°C.

### Typing *var* Gene Expression

To target the group A, B and C *var* genes as well as the cassettes DC1, DC5, DC8, DC13, and DC16, we selected 21 sets of primers from the 42 sets published by Lavstsen *et al*., [20]. The amplification UPS A, B and C groups realized by A1, B1, C1 and C2 primers. For cassettes domains, we have used a subset of primers describe in table 1. Only UM and CM samples were tested with the full set of primers. PAM samples were only tested with *var2csa* specific primers, as this gene has been documented to be the single *Pf*EMP-1 gene expressed in PAM [25], [26]. Quantitative real-time PCR was performed on cDNA using a Rotorgene thermal cycler system (Corbett Research), using the seryl-tRNA synthetase (P90) and fructose-biphosphate aldolase genes endogenous (P61) controls [25], [26]. Reactions were performed in a final volume of 20 μL including 0.1 µl of cDNA, 10 µl of Quantitect SYBR Green PCR Master Mix (Qiagen) and 10 µmol/L primers. Cycling conditions were 50°C for 2 min, 95°C for 10 min, and 40 cycles at 95°C for 15 s and 60°C for 1 min. Data were analyzed using the Rotorgene software 6.0. The cycle threshold (Ct) was set at 0.025. Samples with a mean control gene below 25 were excluded from analysis, to avoid to be in the non-linear amplification range. *Var* genes transcripts abundance was determined as relative quantification with the control gene (ΔCt _var_primer_ = Ct_ var_primer_-Ct _average_control primers_), Transcripts units (Tu) was calculated as Tu = 2 ^(5-ΔCt)^ according to Lavstsen *et al*., [20].

**Table 1:** List of primers used for RT-qPCR [20].

Primer Domain subclass	Domain cassette	UPS Group
**DBLe8**	DC3	A
**DBLb7&9**	DC5	A
**DBLg**	DC5	A
**DBLa1.7**	DC13	A
**CIDRa1.4**	DC13	A
**DBLa1.5/6 (199)**	DC16	A
**DBLa1.5/6 (197)**	DC16	A
**DBLa1.1**	DC1	A
**DBLb12 & DBLb3/5**	DC8	B/A
**CIDRa1.1**	DC8	B
**DBLa_CIDRa**	DC8	B
**DBLg4/6**	DC8	B
**CIDRa1.6**		A
**DBLa1.4**		A
**DBLa2/a1.1/2/4/7**		A
**CIDRa1.7**		A
**DBLb3**		A

The primers targeted the indicated domain subclasses. Some domains occur in domain cassettes and all can be associated to a *Pf*EMP-1 UPS grouping.

### Maturation and Preparation of Samples for Proteomics study

Eleven from CM and 10 parasites from UM were *in vitro* cultured for less than 18 h in order to obtain mature parasite forms (trophozoites and schizonts). Sixty-two placentas positive for *P. falciparum* by rapid diagnostic test were collected, but only 10 were flushed as they showed sufficient parasite density. In these 31 samples (CM, UM and PAM) containing mature forms, parasites were depleted from uninfected erythrocytes over a MACS column [27]; enriched samples contained more than 80% iEs. Samples were lysed according to the method of Fried *et al*., [28]. Briefly, samples were incubated in 10 mM Tris-HCL pH 7.4, 5 mM EDTA, 1% Triton X-100, 1X inhibitor protease (Roche) for 30 min on ice then centrifuged for 30 min at 12,000 g. The lysate was transferred in RIPA buffer with 2% SDS and 1X inhibitor protease (Roche), and stored at −80°C.

To prepare trypsin digestion peptides, 100 µg of proteins were used for UM, PAM and CM samples were reduced with 20 mM DTT during 30 min at 56°C then alkylated with 55 mM of chloroacetamide for 30 min at room temperature. The samples were precipitated in acetone for 2 h at−20°C and centrifuged for 15 min at 14,000 rpm. The lysate was transferred in digestion buffer (50 mM ammonium bicarbonate, 2% rapigest, 20 mM DTT and 1 µg/µl of trypsin). The samples were digested overnight at 37°C. The peptides samples were acidified with 10% of TFA for 2 h at 37°C, and centrifuged 15 min at 14,000 rpm.

### LC–MS/MS Analysis

Analyses were performed using an Ultimate 3000 Rapid Separation liquid chromatographic system (Dionex, The Netherlands) coupled to a hybrid Linear Trap Quadrupole-ORBITRAP Velos mass spectrometer (Thermo Fisher Scientific, San José CA). Briefly, acidified peptides were loaded and washed on a C18 reverse phase precolumn (3 µm particle size, 100 Å pore size, 75 µm i.d., 2 cm length) using a loading buffer containing H_2_O 98%, ACN 2% and TFA 0.1% at 5 µL/minute. Peptides were then separated on a C18 RP analytical column (2 µm particle size, 100 Å pore size, 75 µm i.d., 15 cm length) with a 90-minute gradient from 99% A (ACN 5%, formic acid 0.1% and H_2_O 95%) to 40% B (ACN 80%, formic acid 0.085% and H_2_O 20%).

The LTQ-ORBITRAP mass spectrometer acquired data throughout the elution process and operated in a data dependent scheme as follows: full MS scans were acquired with the ORBITRAP, followed by up to 10 LTQ MS/MS CID spectra on most abundant precursors detected in the MS scan. Exclusion latency was set to 24 seconds for previously fragmented precursors. Mass spectrometer settings were: 1/Full MS with 1.10^6^ Automatic Gain Control (AGC), 3.10^4^ resolutions, 400–2000 mass-to-charge ratio (m/z) range, 1000 ms maximum ion injection time; 2/MS/MS with AGC: 1.10^4^, 200 ms maximum injection time, 2000 minimum signal threshold, 2 Da isolation width. The fragmentation was permitted for precursor with a charge state of 2, 3 or 4.

### Spectra Processing

The software used to generate.mgf files was Proteome discoverer 1.2. The threshold of Signal to Noise for extraction values was 3.

### Database Searching

MS/MS spectra were submitted to mascot (Matrix science) version 2.2 search engine [29]. The precursor mass tolerance was set to 2 ppm and the fragment mass tolerance to 0.45 Da. A filter was applied to the search and the probability of false positive was lower than 5%. The database searched was a concatenation of human and *Plasmodium* sequences from NCBI and their reverse sequences plus the *var* genes of sequences from Vardom (http://www.cbs.dtu.dk/services/VarDom/).

The search parameters were set as follows: trypsin specificity, 2 ppm mass tolerance, 1 missed tryptic cleavage, oxidation (M) was set as a variable modification only those proteins with MOWSE score >40 (*p*<0.05) were accepted as identified.

All samples were compared with MyProMS 2.7.2 software [30] and all *Pf*EMP-1 identified were exported without restriction of score of protein. All peptides from *Pf*EMP-1 identified by LC – MS/MS were positioned on different DBL and CIDR domains of *Pf*EMP-1 from seven *Plasmodium* genomes [12] using Blastp 2.2.24 software. We considered as “correct” the peptides that presented a score of peptides ≥10 with at least six amino-acids. We considered a peptide predictive to a domain if the peptide matched only with that domain subtype (e.g. NTS, DBL, CIDR and ATS) and matched several variants of that domain type (3D7; DD2; IGH; RAJ116; HB3; IT4; PFCLIN). When possible, each peptide was associated with a domain from groups UPS A, B, C and E (VAR2CSA). Several samples presented peptides associated with groups B or C that impaired to distinguish between both groups. We thus considered a combined group (B or C). We considered reliable the identification of a *Pf*EMP-1 protein when it was identified by at least two peptides and a score of protein ≥20.

Correlation between *var* genes transcription level and identified *Pf*EMP-1 proteins.

For ten CM samples, both RT-qPCR and proteomics data were available. We compared if the predominant *Pf*EMP-1 protein identified by LC-MS/MS correlated with the level of transcription of *var* genes from the same UPS group.

### Statistical Analysis

*Var* gene transcription was measured by the relative copy number method, and compared between two groups by the Wilcoxon rank sum test. The mean proportion of peptides identified as associated to UPS groups was compared between the three clinical groups by Kruskall-Wallis test and Wilcoxon rank sum test. Data were plotted using Prism software (version 6; Graphpad). The concordance between genomic and proteomic data has been evaluated by the Cohen’s kappa coefficient. STATA 12 software was used for statistical analyses. P values <0.05 were considered as significant.

## Results

### Patient Samples

Thirty-two children with cerebral malaria (CM), 30 patients with uncomplicated malaria (UM), and 15 pregnant women at delivery were included in the study (Table 2). The average age was three years in the CM group, and 18 years in the UM group, with 14/32 and 12/30 females, respectively. The mean age of the pregnant women was 27 years and six were primigravidae. The mean parasite densities were 216 710, 19 417 and 400 parasites/µl in the CM, UM, and PAM groups, respectively. Nine children with CM died during the first week after hospital admission. These nine children were all referred from another health center, and probably received adequate treatment late after the onset of symptoms.

**Table 2:** Clinical and biological characteristics of patients presenting with malaria in Benin.

	CM	UM	Placental PAM
	(N = 32)	(N = 30)	(N = 15)
**Sex ratio** **(female/male)**	14/18	12/18	15/0
**Age (years)**	3	18	27
**mean (95CI)**	(2; 4)	(12; 25)	(24; 31)
**Parasitemia** **(p/µl)**	216,710	19,417	400
**mean (95CI)**	(47,243; 543,900)	(1,772; 87,798)	(120; 1,580)
**Hemoglobin** **g/dl**	5.6	11.6	–
**mean (95CI)**	(4.0; 7.9)	(10.4; 12.9)	–
**Blantyre score**	2	–	–
**mean (95CI)**	(2; 2)	–	–
**Number of** **deaths**	9	0	0

Cerebral malaria (CM), uncomplicated malaria (UM), or pregnancy-associated malaria (PAM).

### *Var* Transcript Analysis

**Figure 1:**
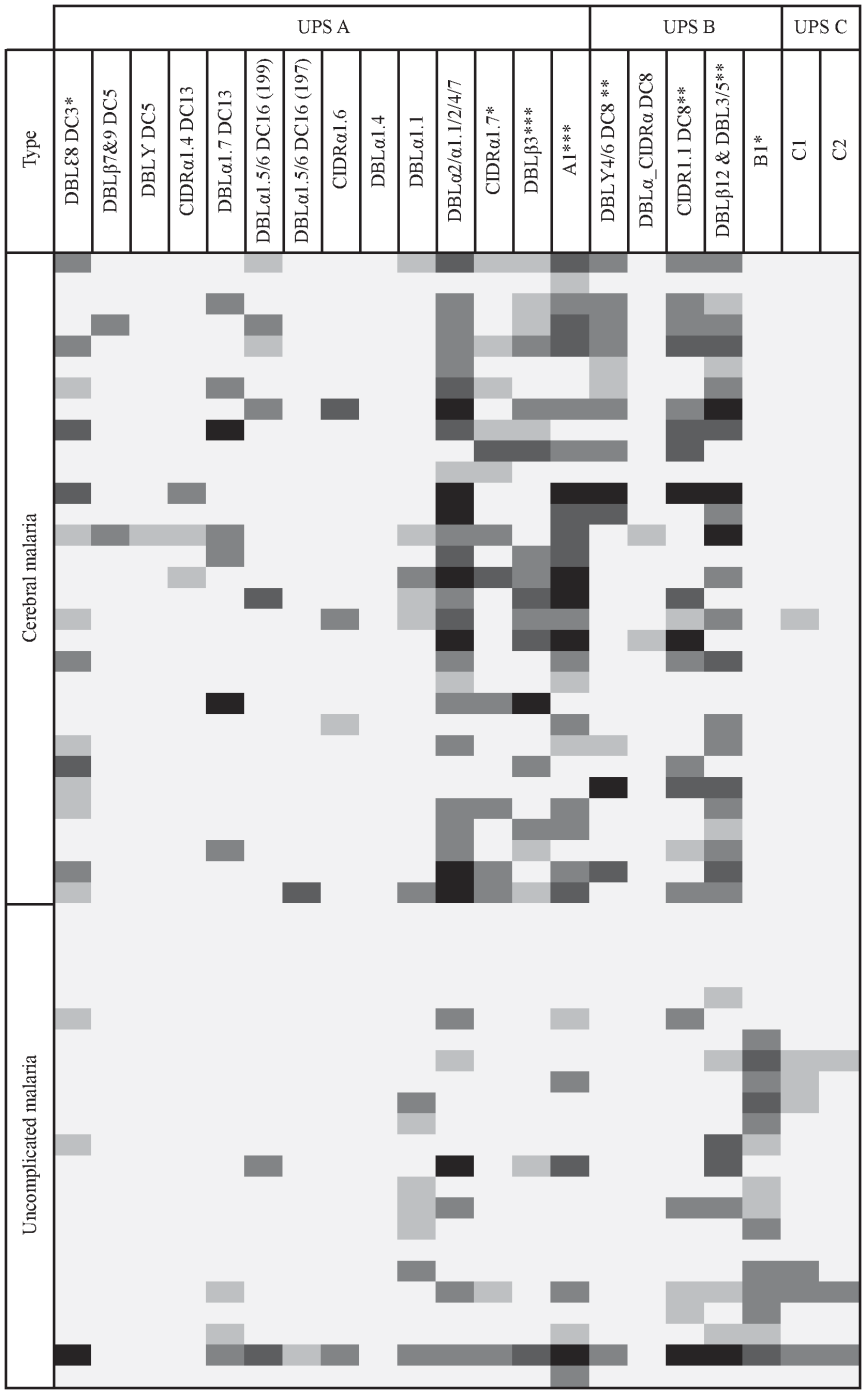
*Var* type transcription in samples from patients with cerebral and uncomplicated malaria in Benin. White boxes show transcript units (Tu) <2; light-grey box: 2<Tu<5; mid-grey box: 5<Tu<20; dark-grey box: 20<Tu<50 and black box: Tu>50. Statically significant higher transcript levels in patients with cerebral malaria than in patients with uncomplicated malaria is indicated by (*) p≤0,05; (**) p≤0,01; (***) p≤10^−3^. Statistically significant higher transcript level among patients with uncomplicated malaria compared to patients with malaria cerebral is indicated as (+) p≤10^−3^.

Thirty-one CM samples and 22 UM samples were analyzed by RT-qPCR with a set of 21 primers [20] targeting group A-C or specific domain cassettes 2, 3, 5, 8, 13 and 16 (Table 1). Figure 1 shows the transcript levels in individual patients. Primers (A1, B1, C1 & C2) clearly showed that group A *var* transcription is significantly higher in CM samples (median transcription units (interquartile range IQR) = 11.2 (1.7–27.2) *vs*. 1.0 (1.0–4.1); *P* = 0.003) whereas group B *var* transcript levels with B1 primer was higher in UM than in CM (4.35 (1.2–12.8) *vs.* 1.0 (1.0–1.0); P<10^−3^). The group C targeted with primer C1 and C2 was similar in two groups CM and UM (1.0 (1.0–1.0) in CM *vs.* 1.0 (1.0–2.0) in UM and 1.0 (1.0–1.0) in CM *vs.* 1.0 (1.0–1.0) in UM, respectively).

All the primer pairs targeting DC8 (domains CIDRα1.1, DBL4/6 and DBLβ12), showed increased transcript levels in CM than in UM samples (2.6 (1.0–15.4) *vs.* 1.0 (1.0–1.7); *P* = 0.0036; 1.0 (1.0–8.7) *vs.* 1.0 (1.0–1.0); *P* = 0.005 and 7.6 (1.3–20.1) *vs.* 1.0 (1.0–2.7); *P* = 0.002, respectively) and a higher prevalence of transcription (as defined with at least one DC8 primer) in CM than in UM (41.9 (24.5–60.9) *vs*. 4.3 (1.1-21.9); *P* = 0.0003). The DBLa1.7 primer pair targeting DC13 showed increased transcript levels in CM compared to UM samples (22.5 (9.6–41.1) *vs*. 4.3 (1.1–21.9); *P* = 0.047). Primers DBLb3 targeting a subset of *var* genes including genes encoding DC8 and DC13 *Pf*EMP-1 also showed significantly higher transcript levels in CM *vs.* UM samples (2.5 (1.0–9.5) *vs.* 1.0 (1.0–1.0); *P = *0.0001.

*Var2csa* transcripts were found at significantly higher levels in PAM than in CM or UM samples (173.2 (134.5–211.9) *vs.* 1.17 (1–4.5) *vs.* 5.45 (1.0–31.1); *P* = 0.0001 for overall comparison, all *P<*10^−3^ for paired [2×2] comparisons, data not shown).

### Proteomics Data

To investigate *Pf*EMP-1 protein expression 11 CM, 10 UM and PAM protein lysates were prepared for LC–MS/MS analysis. Identified peptide sequences were annotated using a database containing human and *Plasmodium* genomes as well as additional available *var* gene sequences. Due to the extensive sequence variation of *Pf*EMP-1, it is expected that the extracted *Pf*EMP-1 molecules would not match 100% to the reference database sequences. Thus, the search parameters were initially varied to test the signal to noise ratio of the matched peptides to *Pf*EMP-1 and human protein database. The analysis first focused on peptides sequences that have been detected in mass spectrometry in the aim to detect all these peptides without any limit of identification score for protein. This also allows to identify major domains and domain cassettes matching with these peptides. Then, we considered only proteins identified with at least two peptides.

The analysis on peptides without any restriction of score allowed to detect 548 peptides matching only with *Pf*EMP-1 proteins; among those, the 440 peptides with a minimal score 10 were retained for analysis. These peptides matched with at least one of the 399 *Pf*EMP-1 proteins from the *Pf*EMP-1 database [12]. 254 peptides were present only in CM samples, 98 only in the PAM samples, 53 only in UM samples, and 35 were present in at least two of the groups. The detected peptide sequences were then mapped back to all known DBL and CIDR domains from seven sequenced *P. falciparum* genomes [12] to investigate if they represented sequence traits specific to certain domain subtypes or *Pf*EMP-1 groups. To avoid non-informative peptides (matching with several different *Pf*EMP-1 domains) and/or poorly predictive peptides, we added selection criteria defined in the materials and methods section. The 440 peptides have been annotated as specific to a *Pf*EMP-1 domain type; 196 peptides being predicted to belong to one *Pf*EMP-1 group or one DC. Among these peptides, 107 were specific to CM samples, 56 to PAM samples, and 20 to UM samples while 13 peptides were found in two groups (table S1). Peptides predicted to origin from DC8 *Pf*EMP-1 were more frequently found among CM samples than in UM and PAM isolates (0.04 (0–0.3) *vs.* 0 (0–0), *P = *0.051 and 0.04 (0–0.3) *vs.* 0 (0–0), *P = *0.045, respectively). Conversely, cassettes DC13 and DC16 were found with similar frequency in isolates from the three clinical groups (DC13: 0 (0–0) in CM, 0 (0–0) in PAM, and 0 (0–0) in UM, *P = *0.273; DC16: 0 (0–0) in CM, 0 (0–0) in PAM, and 0 (0–0) in UM, *P = *0.440, respectively).

We identified at least one *Pf*EMP-1 protein in 10/11; 6/10 and 9/10 isolates from CM, UM, and PAM, respectively, with at least two peptides that yielded a score >20. A total of 104 *Pf*EMP1 proteins were predicted (table S2). VAR2CSA variants were only identified in PAM parasite, with a highest score of 303 with six peptides and 1.7% sequence coverage. Sixteen *Pf*EMP1 proteins or variants DBL domains were identified in parasites from at least two groups (CM, UM and PAM), while 72 *Pf*EMP-1 proteins were identified specifically in CM isolates (17 proteins from group A, 29 from group B, 12 from group C, and 14 proteins with no current annotation). The protein IT4var07, that has been implicated in CM [22], was predicted in 4/11 isolates from CM patients with a maximum score of 421 with five peptides and 2.2% of coverage sequence. Twenty-four *Pf*EMP-1 proteins were identified only in UM samples (4 proteins from group A, 11 from group B, 2 from group C, and 7 proteins with no annotation).

**Figure 2:**
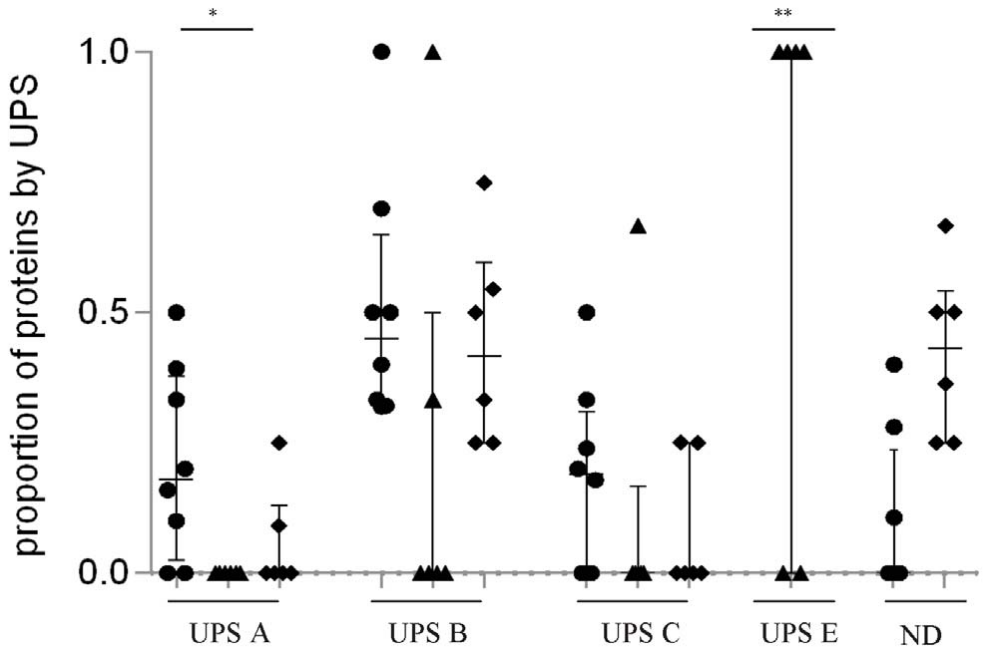
Proportion of proteins identified by LC-MS/MS in parasite samples in Benin, by clinical group. Cerebral malaria (CM, circle), pregnancy-associated malaria (PAM, triangle), and uncomplicated malaria (UM, diamond). Each data point represents the proportion of proteins associated to each UPS group among all *Pf*EMP-1 proteins, for one patient. Bars indicate median with interquartile range. (*) shows a difference between CM vs PAM *P* = 0.017. (**) shows a difference between PAM vs CM and vs UM *P* = 0.0085, *P* = 0.0190 respectively.

We compared the proportion of proteins associated with each group among all identified *Pf*EMP-1 proteins, by isolate and by clinical group (figure 2). The proportion of B and C group proteins was similar in CM, PAM, and UM groups (median distribution of group B and C (IQR) 0.32 (0.45–0.55) in CM *vs.* 0 (0–0.25) in PAM *vs.* 0.42 (0.27–0.53) in UM; *P* = 0.139 and; 0.19 (0–0.26) in CM *vs.* 0 (0–0) in PAM *vs.* 0 (0–0.19) in UM; *P* = 0.422, respectively). Nevertheless, the proportion of group B proteins tended to be higher in CM than in PAM groups (*P* = 0.06).

The number of VAR2CSA (UPSE) proteins differed between the three clinical groups (0 (0–0) in CM *vs.* 1 (0.25–1) in PAM *vs.* 0 (0–0) in UM; *P* = 0.004), being found more frequently in PAM than in the other two clinical groups (both <*P* = 0.02). Similarly, the number of UPS A *Pf*EMP1 proteins differed among clinical groups (0.18 (0.075–0.35) in CM *vs.* 0(0–0) in PAM *vs.* 0 (0–0.07) in UM; *P* = 0.017), being found more frequently in CM isolates than in PAM (*P* = 0. 010) and UM isolates (*P* = 0.081).

**Figure 3:**
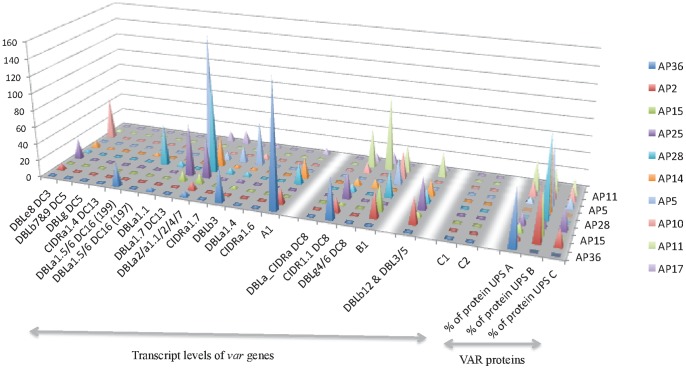
*Var* gene transcript levels and *Pf*EMP-1 proteins identified in the samples collected from patients with cerebral malaria. Each line corresponds to one sample, the right axis shows *var* genes transcription levels for each sample and the left axis shows the proteins identified by LC-MS/MS.

Finally, genomics and proteomics data were compared showing a strong concordance of the levels of *var* gene transcripts from each *var* group (A-C) and the identified *Pf*EMP-1 proteins in CM samples (figure 3) with a Cohen’s kappa coefficient of 0.60. For example, in isolate AP36 a high level of group A transcripts and only *Pf*EMP-1 proteins of the group A were identified by RT-qPCR and LC-MS/MS, respectively. Likewise, in isolate AP15, primarily group B *var* genes and *Pf*EMP1 of UPS B proteins were identified. In addition, in all samples but AP36, we observed *Pf*EMP-1 proteins associated to group B and C, showing that concomitant expression of multiple *Pf*EMP-1 proteins (either related to polyclonal infections or to clonal phenotypic variations) is frequent in CM samples. This phenomenon was less pronounced in the genomics data, as our RT-qPCR approach targets already defined regions corresponding to the set of primers used.

## Discussion

This study aimed to characterize the expression of *var* genes and *Pf*EMP-1 proteins in parasites isolated from children suffering from severe cerebral malaria or uncomplicated malaria as well as women diagnosed with PAM in Cotonou, Benin. We used RT-qPCR and mass spectrometry techniques to produce the first study to associate genomic data on *var* genes with proteomic data on *Pf*EMP-1 expression in *P. falciparum* patients isolates.

As expected ([14], [31]), transcription of group A *var* genes were associated with parasites from children with severe malaria. In addition, using a set of 21 primer pairs targeting *var* transcripts encoding specific *Pf*EMP-1 domain cassettes, parasites from CM patients were found to have higher transcript levels of DC8 and DC13 encoding *var* transcripts *Pf*EMP-1 than parasites from UM patients. This finding on parasite isolated from children from West Africa corroborate previous observations made using the same technology on parasites from Tanzania, East Africa [20] and thus suggest that the association of DC8 *Pf*EMP-1 and severe childhood pathogenesis is not geographically-restricted. Such finding is of utmost importance for future studies of pathogenesis and development of vaccine constructs.

The proteomics approach is particularly difficult to apply to investigations of *P. falciparum* proteins expressed on the membrane of the erythrocyte. Firstly, parasite proteins represent a tiny minority among the far more abundant erythrocyte proteins, and the identification of such rare proteins requires a very efficient MS protocol. Secondly, these variant parasite surface-expressed proteins are characteristically poorly soluble with a very long and highly variable extracellular domain. The use of a 50 cm-long HPLC column allowed a better separation of the peptides, and data analysis was optimized by performing three searches against the Human, *Plasmodium* genomes and *Pf*EMP-1 database of 399 known *Pf*EMP-1 sequences using different settings of 10 and 20 parameters. Next, all predicted *Pf*EMP-1 peptide sequences were annotated with a domain subtype and *Pf*EMP-1 group only if the sequence uniquely matched more than one domain of the said type.

As shown in figure 2, identifying a unique *Pf*EMP-1 variant associated with cerebral malaria, as opposed to PAM, remains a difficult quest. In comparison with parasites from the other clinical groups, proteins and peptides identified by LC-MS/MS in CM isolates are preferentially associated with *Pf*EMP-1 variants encoded by group A *var* genes, confirming our transcriptomic data, and in line with other studies [17]–[19], [21]. Although the identified group B/A genes were few, they tended to be more frequent in CM than in UM and PAM samples. The DC8 (CIDRα1.1 and DBLβ12 domains) is highly expressed in CM samples and is associated with group B/A genes. Identifying a single *Pf*EMP-1 that is uniquely characteristic of CM is unlikely. Conversely, the identification of specific domain cassettes expressed during CM appears to be a real alternative. Thus, we confirmed the over-expression of DC8 in CM parasite samples from West Africa, suggesting that this particular cassette is associated with CM right across sub-Saharan Africa.

Genomics and proteomics data showed a good correlation between the over-expressed *var* genes patterns and the *Pf*EMP-1 proteins identified by LC-MS/MS (figure 3). A unique *Pf*EMP-1 protein was more difficult to identify in the CM samples, suggesting that more than one *Pf*EMP-1 protein is likely involved in the pathogenesis of CM. The expression of more than one *Pf*EMP-1 protein highly complicates genomic and proteomic studies.

In conclusion, our data show that parasites isolated from Beninese children with CM preferentially express *Pf*EMP-1 variants encoded by UPS A and by UPS B/A *var* genes. The protein products of these genes are characterized by their content of the recently described DC13 and DC8 components. Our findings extend observations of these syndrome-specific associations from East to West Africa, providing support for the concept that these *Pf*EMP-1 variants represent virulence factors in various settings. They thus serve to strengthen the evidence base necessary to justify focusing on these variants in the search for an appropriate *Pf*EMP-1 candidate that could form the basis for development of an anti-malarial vaccine.

## Supporting Information

Table S1 **List of peptides predictive of domain subtype, UPS group, Domain Cassette (DC) in parasite samples.** Cerebral malaria (CM), uncomplicated malaria (UM), or pregnancy-associated malaria (PAM).
(DOCX)

Table S2 **List of PfEMP1 proteins identified by LC-MS/MS in parasite samples from different malaria syndromes.** Cerebral malaria (CM), uncomplicated malaria (UM), or pregnancy-associated malaria (PAM).
(DOCX)
