# Supplementary material for: Expression of the Domain Cassette 8 Plasmodium falciparum Erythrocyte Membrane Protein 1 Is Associated with Cerebral Malaria in Benin
Source: PLoS One. 2013 Jul 29;8(7):e68368. doi: 10.1371/journal.pone.0068368 (PMC3726661; doi:10.1371/journal.pone.0068368)
Supplement: Table S1 — List of peptides predictive of domain subtype, UPS group, Domain Cassette (DC) in parasite samples. Cerebral malaria (CM), uncomplicated malaria (UM), or pregnancy-associated malaria (PAM). (DOCX) [file pone.0068368.s001.docx]

**Table S1: List of peptides predictive of domain subtype, UPS group, Domain Cassette (DC) in parasite samples.**

Cerebral malaria (CM), uncomplicated malaria (UM), or pregnancy-associated malaria (PAM).

| Type | Accession number | Description | Score of proteins | Peptide data (distinct) Seq | Score of peptide | Domain subtype prediction | Group UPS prediction | DC prediction |
| --- | --- | --- | --- | --- | --- | --- | --- | --- |
| CM | - | RAJ116var08 | 189.94 | SEETTDIDVLYSGK | 82 | CIDRa1.1 | B | DC8 |
| CM | - | IT4var22 | 212.95 | NSNDLLGNILVTAK | 77 | DBLa1 | A | - |
| CM | 124015305 | PfEMP1 | 421.34 | LVNDSFLGDVLLSAK | 70 | DBLb | A | - |
| CM | - | PFCLINvar59 | PfEMP1 | VNNSFLGDVLLAAK | 63 | All DBLb | A | DC1 |
| CM | - | PFCLINvar06 | 141.29 | STDGTNNDPTLTSPYK | 62 | DBLb5 | B | - |
| CM | 124015305 | IT4_var7 | 171,54 | ASDSFLGDVLVAAK | 53 | DBLb | A | - |
| CM | 78039197 | PfEMP1 | 49.53 | AITCNAPTDAYYFVYK | 50 | DBLa1.4/1.6 | A | - |
| CM | - | PFCLINvar68 | 47.36 | AYAQLEESGSR | 47 | DBLb3 | A | DC8 |
| CM | - | IT4var22 | 212.95 | FDEGQVYECGSGIIK | 47 | DBLa1 | A | - |
| CM | 86170340 | PfEMP1 | 89.26 | YQTLYEEAER | 37 | DBLb | A/ B/A | DC8 |
| CM | - | DD2var19 | 67.73 | LLNEEIKK | 31 | DBLz6 | - | DC10 |
| CM | - | DD2var32 | 32.66 | FSGENSPTLEK | 29 | DBLg | A | - |
| CM | - | PFCLINvar75 | PfEMP1 | NVLDVLAQNIR | 28 | NTSA5 | A | - |
| CM | 34525764 | PfEMP1 | 166.98 | LPMDVPQYDVSK | 27 | ATSA | A | - |
| CM | 90193379 | PfEMP1 | 167.28 | NLEAINVHNTK | 25 | DBLa1 | A | DC16 |
| CM | 124805005 | PfEMP1 | 192.56 | HPPHEYEVACK | 25 | DBLb6 | A | DC13 |
| CM | - | DD2var09b | 224.33 | SSLIGNIK | 24 | DBLb | - | DC5 |
| CM | - | DD2var16 | 141,01 | DLLDMIGK | 24 | PfEMP1 | B | - |
| CM | - | PFCLINvar46 | 23.86 | TTSGATPTEIK | 24 | CIDRa3.1 | B or C | - |
| CM | 15991381 | PfEMP1 | 59.82 | NQEEAAVAVTVK | 23 | CIDRa2 | B or C | - |
| CM | - | DD2var23 | 76.51 | QLSKIIEK | 23 | CIDRa3.1 | B or C | - |
| CM | - | RAJ116var05 | 20.97 | ITEQNIK | 21 | CIDRb4 | A | - |
| CM | - | IGHvar22 | 20.53 | NIYDNLEK | 21 | DBLz2 | - | - |
| CM | 323392499 | PfEMP1 | 20.31 | GKDLYLGGNSK | 20 | DBLa0.7 | B | - |
| CM | - | IGHvar07 | 32.19 | NGITSSGTENK | 20 | DBLb8/12 | - | DC8 |
| CM | 34525764 | PfEMP1 | 166.98 | INHSFLGDVLLAAK | 20 | DBLb3 | A | - |
| CM | 124505153 | var (3D7-varT3-2) | 64.35 | NDFEDEKDK | 20 | DBLa2 | - | DC8 |
| CM | - | DD2var31 | 19.4 | CNNDCKCFLK | 19 | CIDR2/3 | B or C | - |
| CM | - | IGHvar01 | 22.76 | DAKDLLDR | 19 | NTSB3 | B | - |
| CM | - | IGHvar31 | 71.57 | CNGSNPSEKEDINK | 19 | CIDRg5 | B or C | - |
| CM | - | DD2var32 | 92.37 | IGTTWQTGRGIQIK | 19 | DBLb1/7 | A | DC5 |
| CM | - | IGHvar09 | 76.26 | TPSLTESHNSAR | 19 | NTSA | A | - |
| CM | 124015277 | PfEMP1 | 19.21 | CPPCGVKR | 18 | CIDRa5/a3.1/a3.2 | B | - |
| CM | 197252529 | PfEMP1 | 17.92 | MAPGSGDPQDGADK | 18 | NTSB3 | B | - |
| CM | - | PREICHvar52 | 24.33 | GCLENNNGNR | 17 | CIDRa3.1/3.2 | B or C | - |
| CM | 156027219 | PfEMP1 | 17.07 | GRDLFLGNDVEK | 17 | DBLa0 | B or B/A | - |
| CM | 86170340 | PfEMP1 | 49.38 | YSYDELNNAK | 17 | PfEMP1 | B/A | DC8 |
| CM | 124512172 | PfEMP1 | 22.7 | SGDTKVNCNR | 16 | CIDRb1 | B or C | - |
| CM | - | DD2var19 | 23.79 | ACPLCGVNGPKGK | 16 | CIDRa2 | B or C | - |
| CM | 34525764 | PfEMP1 | 16.02 | RDNGNAESELK | 16 | DBLg6 | A/ B | DC8 |
| CM | 343175984 | PfEMP1 | 15.94 | CTKSSGGKPGK | 16 | DBLa0 | B or C | - |
| CM | 124805350 | PfEMP1 | 15.81 | VLASLTNCYKCDK | 16 | DBLpam2 | E | DC2 |
| CM | - | PFCLINvar67 | 15.64 | ELNSCINNPK | 16 | CIDRa2/3 | B or C | - |
| CM | 323393345 | PfEMP1 | 15.11 | YEGNSIER | 15 | DBLa0 | B or C | - |
| CM | 154359876 | VAR2CSA | 15.02 | MDSKTTIAEK | 15 | NTSpam | E | DC2 |
| CM | 323394280 | PfEMP1 | 14.92 | YQDDPNK | 15 | DBLa1.7 | A | DC13 |
| CM | - | RAJ116var08 | 14.87 | LYEELVEACGSCK | 15 | (DBLb12) | A | DC8 |
| CM | - | DD2var42 | 14.84 | NIDFTKSGDEK | 15 | DBLa1 | A | - |
| CM | 78041701 | PfEMP1 | 14.68 | SKYNGDTTDFFK | 15 | DBLa0 | B | - |
| CM | - | IGHvar36 | 23.08 | LENSENSLFK | 15 | CIDRg7/g12 | B | - |
| CM | - | IGHvar37 | 14.42 | STLESLTDEEIR | 14 | DBLa0 | B or C | - |
| CM | 19109016 | PfEMP1 | 14.38 | IYNEVTTNGK | 14 | DBLa0 | B or C | - |
| CM | - | PFCLINvar71 | 40.33 | AITCSTPR | 14 | DBLz3 | - | DC12 |
| CM | 78041989 | PfEMP1 | 14.01 | GEAQANKYCR | 14 | DBLa0.16/0.15 | B or C | - |
| CM | - | HB3var35 | 39.04 | IDFNDPK | 14 | DBLd1 | B | - |
| CM | - | IGHvar17 | 13.79 | GIFSNDPK | 14 | DBLz6 | - | DC10 |
| CM | 124807212 | PfEMP1 | 13.72 | SQEGVLQTK | 14 | DBLd1 | B | - |
| CM | 19879270 | PfEMP1 | 43.92 | DGQNFYQLR | 13 | DBLa0 | B or C | - |
| CM | - | HB3var08 | 23.14 | NQVEVGADSKK | 13 | CIDRa2.7 | B | - |
| CM | 197252773 | PfEMP1 | 13.32 | KSAEAQDR | 13 | DBLa2 | B/A | DC8 |
| CM | 34525752 | PfEMP1 | 13.24 | EECIGKNK | 13 | DBLa1.5 | A | DC16 |
| CM | 261863946 | PfEMP1 | 13.01 | NNDNIWTDLVK | 13 | DBLe10 | E | - |
| CM | 124806691 | PfEMP1 | 12.79 | IYEGLTGGVKER | 13 | DBLa0 | B or C | - |
| CM | - | DD2var23 | 12.72 | GRDQLESK | 13 | DBLa0 | B or C | - |
| CM | 124505645 | PfEMP1 | 24.37 | YEGDSIK | 13 | PfEMP1 | B or C | - |
| CM | 270484054 | PfEMP1 | 12.56 | ISIEQVREYR | 13 | DBLa2/1.7 | A or B/A | - |
| CM | 124015295 | PfEMP1 | 12.4 | TNQICCDELPESK | 12 | PfEMP1 | - | - |
| CM | - | PFCLINvar55 | 12.38 | GAPGTAKDGVR | 12 | CIDRa2 | B or C | - |
| CM | - | IGHvar36 | 12.38 | NGAPDNSNR | 12 | DBLa0 | B or C | - |
| CM | - | RAJ116var08 | 189.94 | IIEEILR | 12 | CIDRa1.1 | B/A | DC8 |
| CM | - | HB3var21 | 12.21 | ITLEHSNDSR | 12 | PfEMP1 | B | DC1 |
| CM | 124015263 | PfEMP1 | 12.2 | DEEEAEAGVTDNQKK | 12 | CIDRa3.2 | B or C | - |
| CM | 7109146 | PfEMP1 protein | 12.19 | NQVWEAITCGALPK | 12 | DBLa1.1 | A | DC1 |
| CM | - | HB3var20 | 12.06 | KDINFCVLQDNK | 12 | CIDRa2 | B or C | - |
| CM | 78032715 | PfEMP1 | 11.85 | TVCSEGTSPTQGK | 12 | DBLa0 | B or C | - |
| CM | - | HB3var22 | 11.8 | NLPDDVKDECECK | 12 | DBLe2 | - | DC7 |
| CM | 251831552 | PfEMP1 | 11.59 | EIHSEVTNGK | 12 | DBLa0 | B or C | - |
| CM | 124805331 | PfEMP1 | 64.06 | QKDMIETGMPPEMALK | 12 | CIDRa2.2 | B | - |
| CM | - | RAJ116var30 | 72.97 | CDCFLKWVNEK | 11 | CIDRa2/3 | B or C | - |
| CM | - | HB3var13 | 40.21 | KLEDAIK | 11 | DBLa0 | B or C | - |
| CM | - | RAJ116var09 | 32.21 | QLGNCINNNTNDNR | 11 | CIDRa2 | B or C | - |
| CM | - | RAJ116var31 | 11.26 | SSSAADGGDSNNK | 11 | DBLa0.15 | B or C | - |
| CM | - | DD2var45 | 24.27 | NCNGKCDCFLK | 11 | CIDRa3.1 | B or C | - |
| CM | 19108685 | PfEMP1 | 11.16 | ACSGGKTWTNK | 11 | DBLa0.9 | B | DC20 |
| CM | 124805343 | PfEMP1 | 11.16 | SSVDNKCIMQTNNQK | 11 | CIDRa5 | B | - |
| CM | 90193303 | PfEMP1 | 11.14 | IVVGDETHK | 11 | NTSA6 | A | - |
| CM | - | DD2var19 | 11.01 | IANEAALKR | 11 | NTSB3 | B | - |
| CM | 323392769 | PfEMP1 | 63.53 | NEEDAVQKGLK | 11 | DBLa1.5/1.2 | A | - |
| CM | - | IGHvar39 | 20.71 | QWKQMEQK | 11 | DBLb3 | A | - |
| CM | 124807212 | PfEMP1 | 10.91 | TCGSGNNATQAKDK | 11 | DBLa0 | B or C | - |
| CM | - | PFCLINvar59 | 53.09 | NNHPAIQGNHK | 11 | DBLb5 | B | - |
| CM | - | RAJ116var02 | 10.78 | ENINGACMPPRR | 11 | DBLg8 | A | DC1 |
| CM | 124511806 | PfEMP1 | 10.75 | KSDGSGGWEK | 11 | CIDRa2.1 | B or C | - |
| CM | - | IGHvar33 | 23.94 | MVPQSRSGGGK | 11 | NTSB3 | B | - |
| CM | 78041313 | PfEMP1 | 10.61 | EIYDNLMEDLKK | 11 | DBLa0.11 | B | - |
| CM | 197252361 | PfEMP1 | 10.54 | QAEKDASK | 11 | NTSA2 | A | - |
| CM | 124805350 | PfEMP1 | 10.53 | NNDSNGLPK | 11 | DBLpam3 | E | - |
| CM | - | RAJ116var21 | 18.35 | EVDGTGASGDAKK | 11 | NTSB3 | B | - |
| CM | - | RAJ116var24 | 11.17 | KEEEWK | 10 | CIDRa3.1 | B or C | - |
| CM | - | RAJ116var33 | 11.05 | EEDACDIMDKIYGTSNNIK | 10 | DBLg | B | - |
| CM | - | PFCLINvar29 | 62.78 | MEQIIEK | 10 | CIDRa2.4 | B | - |
| CM | - | HB3var16 | 18.98 | DKLNSCINNK | 10 | CIDRa2.2 | B | - |
| CM | 86374140 | PfEMP1 | 10.15 | TACGTGTPTPNK | 10 | PfEMP1 | B or C | - |
| CM | - | RAJ116var06 | 64 | THGPNAARR | 10 | DBLz4 | - | DC9 |
| CM | - | DD2var22 | 41.22 | YEGASIVEK | 10 | DBLa1 | A | - |
| CM | - | PFCLINvar25 | 10.07 | FLQEELK | 10 | CIDRa2 | B or C | - |
| CM | 26985360 | varPAM | 10.06 | QAYDQANYR | 10 | DBLb1 | A | DC1 |
| COMMON | 124805350 | PfEMP1 | 62.65 | DTFNTSSGDTFTNK | 63 | ATSpam1 | E | - |
| COMMON | - | HB3var50 | 30.18 | LLNEEIK | 30 | DBLz6 | - | DC10 |
| COMMON | 323393477 | PfEMP1 | 22.33 | DXGSTICTVLAR | 22 | DBLa0 | B or C | - |
| COMMON | - | HB3var48 | 21.1 | IAEKNVEPQLK | 21 | DBLz4 | B | DC9 |
| COMMON | 14578876 | PfEMP1 | 19.28 | DNLEDKLK | 19 | DBLz4 | B | DC9 |
| COMMON | 124805343 | PfEMP1 | 16.19 | KENELFGK | 16 | ATSB | B or C | - |
| COMMON | 124505551 | PfEMP1 | 15.91 | QFYEEFK | 16 | DBLa0 | B or C | - |
| COMMON | 323392627 | PfEMP1 | 15.66 | TACSNDQSWANHNCR | 16 | DBLa0 | B or C | - |
| COMMON | 323394629 | PfEMP1 | 15.64 | NDVWKAITCEVK | 16 | DBLa0 | B or C | - |
| COMMON | - | IT4var46 | 27.71 | NSDGNNSDQIEK | 13 | CIDRa2 | B or C | - |
| COMMON | - | DD2var36 | 12.87 | IFLQIFFMDKIK | 13 | CIDRa2.1 | B or C | - |
| COMMON | - | RAJ116var09 | 11.45 | QLGNCINNNTNDNRCK | 11 | CIDRa2.9/2.5 | B or C | - |
| COMMON | 323394430 | PfEMP1 | 46.41 | GEDLYLGNPQESTQRK | 11 | DBLa0 | B or C | - |
| PAM | 154359888 | VAR2CSA | 259.78 | YKDLYQQENETSSSSK | 74 | DBLpam1 | E | DC2 |
| PAM | 154359888 | VAR2CSA | 179.35 | DLYQQENETSSSSK | 67 | DBLpam1 | E | DC2 |
| PAM | 154359874 | VAR2CSA | 106,5 | LNEIFGSSNTNNIDTK | 63 | DBLepam4 | E | DC2 |
| PAM | - | PFCLINvar72 | 255,26 | GIEGEMWGAVR | 61 | DBLpam3 | E | DC2 |
| PAM | 154359886 | VAR2CSA | 127,61 | SFADIADIIR | 60 | DBLpam1 | E | DC2 |
| PAM | 154359888 | VAR2CSA | 259.78 | ETELLYEYHDK | 58 | DBLpam3 | E | DC2 |
| PAM | - | IGHvar41 | 330,67 | QDFIEQYLQNIQK | 53 | ATSpam1 | E | DC2 |
| PAM | 261863956 | PfEMP1 | 54,56 | NLFLNIDPSK | 48 | DBLe10 | E | DC2 |
| PAM | 154359888 | VAR2CSA | 259.78 | SNDLLIK | 47 | DBLpam1 | E | DC2 |
| PAM | 154359886 | VAR2CSA | 199,04 | TVGSGAENVNAWWK | 46 | DBLpam3 | E | DC2 |
| PAM | - | IGHvar41 | 246,37 | GSDMLTNIQFK | 46 | DBLepam5 | E | DC2 |
| PAM | 154359874 | VAR2CSA | 127,61 | YSFADIGNIIK | 44 | DBLe10 | E | DC2 |
| PAM | 154359888 | VAR2CSA | 179.35 | GNDLVHDEYTK | 41 | DBLpam4 | E | DC2 |
| PAM | - | HB3var2csaA | 138.26 | GNDLVHDEYTK | 41 | DBLpam4 | E | DC2 |
| PAM | 154359874 | VAR2CSA | 351,3 | YSFADYGDLIK | 40 | DBLpam2 | E | DC2 |
| PAM | 154359874 | VAR2CSA | 340,94 | GSFYDLEDIIK | 39 | DBLepam4 | E | DC2 |
| PAM | 154359888 | VAR2CSA | 260,7 | NIGSDTFEFLIK | 39 | CIDRpam | E | DC2 |
| PAM | 154359874 | VAR2CSA | 255,26 | EWGEQFCIER | 37 | DBLpam3 | E | DC2 |
| PAM | 154359888 | VAR2CSA | 179.35 | GFCHAVQR | 37 | DBLpam3 | E | DC2 |
| PAM | 154359874 | VAR2CSA | 298,54 | QNLIDDMER | 36 | DBLpam1 | E | DC2 |
| PAM | - | HB3var2csaA | 138.26 | STNNVEELIK | 35 | DBLpam1 | E | DC2 |
| PAM | 31323024 | var2csa | 32.04 | TYDGFISPGK | 32 | DBLpam3 | E | DC2 |
| PAM | 154359874 | VAR2CSA | 255,26 | NSFYDYEYIIK | 31 | DBLepam5 | E | DC2 |
| PAM | - | HB3var2csaA | 268,65 | GVTDINFDTK | 27 | DBLpam2 | E | DC2 |
| PAM | - | HB3var2csaA | 138.26 | NNPYSAEYVTK | 26 | DBLpam1 | E | DC2 |
| PAM | - | PREICHvar95 | 24.22 | EVKDQYDEQK | 24 | CIDRa5 | B or C | DC17 |
| PAM | 270484592 | PfEMP1 | 23.09 | GRDQLEENLK | 23 | DBLa0.8/0.6 | B or C | - |
| PAM | 323393238 | PfEMP1 | 20.56 | NLEEIDPK | 21 | DBLa0 | B or C | - |
| PAM | - | IGHvar37 | 18.25 | QTCVGGSPTQGK | 18 | DBLa0 | B or C | - |
| PAM | - | IGHvar35 | 18.1 | LASASYFR | 18 | DBLa0 | B or C | - |
| PAM | 78039925 | PfEMP1 | 16.11 | IQLEEELKR | 16 | DBLa0 | B or C | - |
| PAM | 124504675 | PfEMP1 | 15.74 | LGGLCGNGAKK | 16 | CIDRa2.4 | B | - |
| PAM | - | IT4var44 | 15.65 | GKHDSLEK | 16 | CIDRb6 | B or C | - |
| PAM | 156027075 | PfEMP1 | 15.57 | HTCGTGKPTNEK | 16 | DBLa0.8 | B or C | - |
| PAM | 90193285 | PfEMP1 | 14.83 | EDRCPCLLR | 15 | DBLa1 | A | - |
| PAM | - | HB3var50 | 14.69 | QSPPAGDGGGVGR | 15 | DBLa0.8 | B or C | - |
| PAM | - | RAJ116var09 | 14.55 | QQEWDAIK | 15 | CIDRa2.9/a3.2 | B or C | - |
| PAM | 197252575 | PfEMP1 | 14.15 | ETNQGTASQLCTVLAR | 14 | DBLa0 | B or C | - |
| PAM | - | PREICHvar85 | 13.99 | KLSGFCK | 14 | CIDRa5 | - | DC17 |
| PAM | - | IGHvar26 | 13.78 | KDNGNAENELK | 14 | DBLg6 | B/A | DC8 |
| PAM | 323394473 | PfEMP1 | 13.72 | AAEARYK | 14 | DBLa0 | B or C | - |
| PAM | 197252397 | PfEMP1 | 13.7 | TNPCLNRSSVR | 14 | DBLa0 | B or C | - |
| PAM | - | IT4var26 | 13.42 | NTEEDKENNVSAEEIDLINK | 13 | CIDRa3.3 | B or C | - |
| PAM | - | HB3var33 | 34.56 | KNGAEELQK | 13 | DBLa0 | B or C | - |
| PAM | 18621273 | PfEMP1 | 12.27 | TDDKYFR | 12 | DBLa0 | B or C | - |
| PAM | 124511794 | PfEMP1 | 12.1 | DTNGQEGDKK | 12 | CIDRb5/6 | B or C | - |
| PAM | 323392962 | PfEMP1 | 12.03 | DVTRGSNEQALQER | 12 | DBLa0 | B or C | - |
| PAM | - | HB3var22 | 11.52 | ELLDIIGEHIYK | 12 | NTSB3 | B | - |
| PAM | - | PFCLINvar72 | 10.89 | DACINNGK | 11 | DBLpam3 | E | - |
| PAM | 323393269 | PfEMP1 | 10.52 | YEGDLIKTR | 11 | DBLa0 | B or C | - |
| PAM | - | HB3var1csa | 10.4 | ENENAACMPPRR | 10 | DBLg8 | A | DC1 |
| PAM | - | RAJ116var23 | 24.16 | MAPQGGSRGGVGEIDDK | 10 | NTSB3 | B | - |
| PAM | 343766138 | PfEMP1 | 10.38 | YFRNTCAGGK | 10 | DBLa0 | B or C | - |
| PAM | 124805350 | PfEMP1 | 10.1 | SSIANKIEAYLGAK | 10 | NTSpam | E | - |
| PAM | 283831642 | PfEMP1 | 10.05 | SIWNAMLCGHKK | 10 | DBLepam5 | E | - |
| PAM | 31323032 | var2csa | 10 | GQGDKIQGACK | 10 | DBLpam3 | E | - |
| UM | 1517814 | PfEMP1 | 22.47 | DYQSMNDFLK | 22 | DBLa0.18 | B or C | - |
| UM | 124511804 | PfEMP1 | 43.08 | IMKPCTSLDK | 22 | CIDRg5 | B | - |
| UM | - | PREICHvar83 | 21.14 | AQHNILDK | 21 | CIDR2.1 | B or C | - |
| UM | 323393343 | PfEMP1 | 37.2 | ATIXEAITCDVK | 20 | x | B or C | - |
| UM | 90193473 | PfEMP1 | 17.47 | TQSLYLGNLR | 17 | DBLpam2 | E | DC2 |
| UM | 90193217 | PfEMP1 | 16.94 | YEGDCIVDHLPDNEK | 17 | DBLa0.21 | B or C | DC21 |
| UM | 78040883 | PfEMP1 | 15.3 | QLEENFKK | 15 | DBLa0 | B or C | - |
| UM | 124511798 | PfEMP1 | 50.97 | DSIYWRMK | 15 | CIDRa3.1 | B or C | - |
| UM | - | IGHvar24 | 34.88 | IEKTIPAEATEAEEK | 14 | PfEMP1 | A | - |
| UM | - | IT4var26 | 43.58 | YQAQYASSVSSSQMCTMLAR | 12 | DBLa0 | B or C | - |
| UM | 124806691 | PfEMP1 | 11.74 | GASSTSDTSGTNDETK | 12 | DBLa0 | B or C | - |
| UM | 197252495 | PfEMP1 | 28.5 | IYDQLKPEAQKR | 12 | NTSB3 | B | - |
| UM | - | IGHvar30 | 11.06 | NDLMNCINNTNK | 11 | CIDRa1.5/1.6 | A | DC16 |
| UM | 311702421 | PfEMP1 | 10.92 | NCIPEIKK | 11 | PfEMP1 | E | DC2 |
| UM | 323392468 | PfEMP1 | 10.85 | TCGSGNWTKDK | 11 | DBLa0 | B or C | - |
| UM | 254952650 | VAR2CSA | 10.64 | DVIENCNSCKNNLGK | 11 | DBLpam2 | E | DC2 |
| UM | - | HB3var22 | 10.43 | GDPKVIQK | 10 | CIDRa2.2 | B or C | - |
| UM | - | PFCLINvar26 | 35.52 | ELSEKYVDR | 10 | DBLa0 | B or C | - |
| UM | 154359870 | VAR2CSA | 10.27 | GTDMLTNIK | 10 | DBLepam5 | E | DC2 |
| UM | 154359888 | VAR2CSA | 10.02 | GWTTSKESEGENK | 10 | DBLpam1 | E | DC2 |
