# Supplementary material for: Expression of the Domain Cassette 8 Plasmodium falciparum Erythrocyte Membrane Protein 1 Is Associated with Cerebral Malaria in Benin
Source: PLoS One. 2013 Jul 29;8(7):e68368. doi: 10.1371/journal.pone.0068368 (PMC3726661; doi:10.1371/journal.pone.0068368)
Supplement: Table S2 — List of PfEMP1 proteins identified by LC-MS/MS in parasite samples from different malaria syndromes. Cerebral malaria (CM), uncomplicated malaria (UM), or pregnancy-associated malaria (PAM). (DOCX) [file pone.0068368.s002.docx]

**Table S2: List of PfEMP1 proteins identified by LC-MS/MS in parasite samples from different malaria syndromes.**

Cerebral malaria (CM), uncomplicated malaria (UM), or pregnancy-associated malaria (PAM)

| Type | Identifier | Description | Mol weight (kDa) | Groups | Number of samples | Score | Number of peptides | Coverage (%) |
| --- | --- | --- | --- | --- | --- | --- | --- | --- |
| CM | gi\|124015305 | IT4var7 | 397,4 | A3 | 4 | 421 | 5 | 2.2 |
| CM | gi\|15991381 | var1 | 292,1 | C | 3 | 230 | 5 | 1.9 |
| CM | - | PFCLINvar67 | 279,2 | C1 | 3 | 102 | 2 | 0.9 |
| CM | - | DD2var19 | 328,5 | B1 | 3 | 68 | 3 | 1.0 |
| CM | - | IGHvar27 | 350,2 | A1 | 2 | 311 | 6 | 2.1 |
| CM | gi\|124015239 | IT4var11 | 287,3 | B1 | 2 | 288 | 6 | 2.5 |
| CM | - | PFCLINvar06 | 327,6 | - | 2 | 282 | 6 | 2.3 |
| CM | - | RAJ116var06 | 338,3 | B1 | 2 | 253 | 4 | 1.9 |
| CM | - | DD2var36 | 329,0 | C1 | 2 | 244 | 4 | 1.4 |
| CM | gi\|124511800 | PF07_0050 | 238,5 | B3 | 2 | 210 | 4 | 2.3 |
| CM | - | RAJ116var08 | 318,3 | B1 | 2 | 190 | 4 | 1.4 |
| CM | gi\|124505153 | var (3D7-varT3-2) | 246,9 | B1 | 2 | 171 | 3 | 1.4 |
| CM | - | IGHvar09 | 390,3 | A1 | 2 | 169 | 4 | 1.4 |
| CM | gi\|124015307 | IT4var08 | 382,2 | B1 | 2 | 158 | 3 | 1.1 |
| CM | - | IGHvar25 | 246,2 | B1 | 2 | 154 | 4 | 1.8 |
| CM | gi\|86170340 | PFF0010w | 333,5 | - | 2 | 89 | 2 | 0.7 |
| CM | - | IGHvar39 | 391,4 | A3 | 2 | 74 | 2 | 0.6 |
| CM | gi\|124512768 | PF13_0003 | 385,8 | A1 | 2 | 52 | 2 | 0.2 |
| CM | gi\|34525762 | var3 | 266,3 | A | 1 | 294 | 5 | 2.8 |
| CM | - | RAJ116var11 | 341,6 | B2 | 1 | 282 | 4 | 1.7 |
| CM | - | RAJ116var35 | 316,3 | C2 | 1 | 231 | 5 | 1.6 |
| CM | - | DD2var09b | 359,7 | A1 | 1 | 224 | 4 | 1.3 |
| CM | - | IGHvar30 | 420,6 | A3 | 1 | 224 | 4 | 1.2 |
| CM | - | IT4var22 | 375,5 | A3 | 1 | 213 | 4 | 1.5 |
| CM | gi\|124805005 | PF11_0521 | 359,2 | A1 | 1 | 193 | 5 | 1.8 |
| CM | - | HB3var36 | 253,0 | C2 | 1 | 176 | 4 | 2.1 |
| CM | gi\|124775746 | PFB0010w | 196,6 | B1 | 1 | 172 | 4 | 2.1 |
| CM | gi\|90193379 | DBL1α 7G8 | 342,0 | - | 1 | 167 | 3 | 11.8 |
| CM | gi\|34525764 | MC_var4 | 346,6 | - | 1 | 167 | 3 | 1.6 |
| CM | - | DD2var29 | 299,6 | B1 | 1 | 123 | 3 | 0.9 |
| CM | - | PFCLINvar69 | 294,0 | A3 | 1 | 120 | 4 | 1.0 |
| CM | gi\|124803377 | PF11_008 | 345,7 | A1 | 1 | 117 | 3 | 1.2 |
| CM | - | RAJ116var28 | 274,0 | C1 | 1 | 113 | 2 | 1.1 |
| CM | gi\|26985360 | varPAM | 145,2 | - | 1 | 100 | 3 | 1.8 |
| CM | gi\|124512608 | PF08_0106 | 256,0 | B7 | 1 | 97 | 2 | 0.9 |
| CM | - | DD2var18 | 246,8 | B1 | 1 | 91 | 3 | 1.8 |
| CM | - | PFCLINvar68 | 275,2 | A3 | 1 | 86 | 2 | 0.5 |
| CM | - | DD2var23 | 347,4 | B1 | 1 | 77 | 2 | 0.9 |
| CM | - | PFCLINvar41 | 199,9 | C1 | 1 | 73 | 2 | 0.9 |
| CM | - | RAJ116var30 | 276,9 | B1 | 1 | 73 | 4 | 2.0 |
| CM | gi\|124807212 | PFL2665c | 255,4 | B1 | 1 | 73 | 2 | 1.0 |
| CM | gi\|124015247 | IT4var19 | 385,4 | B1 | 1 | 72 | 2 | 0.6 |
| CM | - | IGHvar31 | 255,5 | B1 | 1 | 72 | 2 | 1.0 |
| CM | - | HB3var30 | 245,7 | B6 | 1 | 71 | 2 | 1.3 |
| CM | - | PREICHvar29 | 373,5 | A3 | 1 | 70 | 2 | 5.1 |
| CM | - | IGHvar28 | 331,5 | C1 | 1 | 70 | 2 | 0.6 |
| CM | gi\|323394993 | DBLα | 220,6 | - | 1 | 70 | 2 | 9.4 |
| CM | - | RAJ116var32 | 249,3 | B3 | 1 | 68 | 2 | 0.8 |
| CM | - | PFCLINvar28 | 244,2 | B1 | 1 | 67 | 4 | 1.7 |
| CM | gi\|124015243 | IT4var17 | 341,9 | B1 | 1 | 64 | 2 | 1.0 |
| CM | gi\|124805331 | PFL0005w | 248,6 | B1 | 1 | 64 | 2 | 1.2 |
| CM | gi\|323392769 | DBLα | 199,2 | - | 1 | 64 | 2 | 12.0 |
| CM | gi\|86171918 | PFF1580c | 456,9 | - | 1 | 63 | 2 | 0.6 |
| CM | gi\|124015293 | IT4var05 | 246,5 | C1 | 1 | 63 | 2 | 1.0 |
| CM | - | PFCLINvar29 | 107,6 | B2 | 1 | 63 | 2 | 1.8 |
| CM | - | IGHvar23 | 398,4 | A1 | 1 | 62 | 2 | 0.7 |
| CM | gi\|323393414 | DBLα | 221,7 | - | 1 | 62 | 2 | 9.6 |
| CM | gi\|323394629 | DBLα | 225,0 | - | 1 | 62 | 2 | 11.2 |
| CM | gi\|124505931 | PFA_0765c | 250,6 | - | 1 | 54 | 3 | 1.3 |
| CM | - | PFCLINvar59 | 281,6 | - | 1 | 53 | 3 | 1.1 |
| CM | - | IGHvar26 | 378,8 | A1 | 1 | 49 | 2 | 0.5 |
| CM | - | RAJ116var37 | 335,0 | B3 | 1 | 45 | 2 | 0.5 |
| CM | - | HB3var19 | 247,8 | B1 | 1 | 43 | 2 | 1.0 |
| CM | gi\|124805987 | PFL0935c | 255,5 | B1 | 1 | 43 | 2 | 1.5 |
| CM | - | DD2var22 | 333,1 | A3 | 1 | 41 | 3 | 0.9 |
| CM | - | DD2var41 | 306,4 | C1 | 1 | 39 | 2 | 0.6 |
| CM | - | IT4var63 | 243,6 | B1 | 1 | 35 | 2 | 0.8 |
| CM | - | PFCLINvar64 | 253,1 | C1 | 1 | 35 | 2 | 0.6 |
| CM | - | HB3var25 | 329,7 | C1 | 1 | 33 | 2 | 0.5 |
| CM | - | IGHvar07 | 344,0 | B6 | 1 | 32 | 2 | 0.8 |
| CM | - | PFCLINvar45 | 268,9 | - | 1 | 31 | 2 | 0.7 |
| CM | - | PREICHvar96 | 221,1 | - | 1 | 27 | 2 | 0.7 |
| CM and UM | - | DD2var32 | 406,8 | A1 | 4 vs 3 | 212 | 5 | 1.5 |
| CM and UM | - | DD2var16 | 239,2 | - | 3 vs 2 | 125 | 3 | 1.6 |
| CM and UM | - | IGHvar38 | 254,2 | B1 | 2 vs 5 | 85 | 2 | 0.7 |
| CM and UM | gi\|914919 | MC_var1 | 335,9 | C | 2 vs 2 | 112 | 3 | 1.2 |
| CM and UM | - | HB3var50 | 334,5 | B3 | 2 vs 2 | 105 | 3 | 1.3 |
| CM and UM | - | HB3var13 | 242,1 | B1 | 2 vs 1 | 89 | 3 | 1.1 |
| CM and UM | gi\|34525758 | IT4var2 | 390,2 | A3 | 2 vs 1 | 82 | 3 | 0.8 |
| CM and UM | - | RAJ116var09 | 305,4 | B1 | 2 vs 1 | 53 | 2 | 1.0 |
| CM and UM | gi\|323394710 | DBLα | 218,8 | - | 1 vs 3 | 71 | 2 | 9.6 |
| CM and UM | - | HB3var48 | 290,8 | B1 | 1 vs 3 | 67 | 2 | 0.8 |
| UM and PAM | gi\|124504675 | PFC0005w | 250,5 | B1 | 1 vs 1 | 124 | 4 | 2.4 |
| CM and UM | gi\|124512758 | PF08_0140 | 340,6 | B2 | 1 vs 1 | 119 | 3 | 1.2 |
| CM and UM | - | DD2var31 | 249,0 | B1 | 1 vs 1 | 110 | 3 | 1.2 |
| CM and UM | - | IGHvar34 | 320,3 | C1 | 1 vs 1 | 79 | 3 | 1.1 |
| CM and UM | gi\|323394085 | DBLα | 208,0 | - | 1 vs 1 | 70 | 2 | 10.0 |
| CM and UM | gi\|19879270 | AYO28643 | 301,3 | - | 1 vs 1 | 44 | 2 | 0.6 |
| PAM | gi\|154359888 | VAR2CSA | 296,8 | E | 3 | 303 | 6 | 1.7 |
| PAM | gi\|124805350 | PFL0030c | 355,2 | E | 3 | 177 | 5 | 0.5 |
| PAM | - | HB3var2csaA | 336,5 | E | 2 | 138 | 4 | 1.4 |
| PAM | - | PFCLINvar72 | 383,7 | E | 2 | 126 | 3 | 0,8 |
| PAM | - | HB3var33 | 251,4 | C1 | 1 | 35 | 2 | 0.7 |
| PAM | gi\|124505543 | PFD0995c | 247,6 | C1 | 1 | 31 | 2 | 0.7 |
| PAM | - | PFCLINvar24 | 201,5 | B1 | 1 | 29 | 2 | 0.7 |
| PAM | - | IT4var46 | 371,7 | B1 | 1 | 28 | 2 | 0.7 |
| UM | gi\|323394430 | DBLα | 217,7 | - | 4 | 46 | 2 | 13.7 |
| UM | - | IGHvar21 | 367,5 | B7 | 2 | 87 | 3 | 1.1 |
| UM | gi\|323393477 | DBLα | 239,8 | - | 2 | 53 | 2 | 10.8 |
| UM | gi\|124015253 | IT4var24 | 234,5 | B1 | 1 | 115 | 3 | 1.8 |
| UM | - | PFCLINvar22 | 143,2 | B2 | 1 | 53 | 2 | 1.4 |
| UM | - | PREICHvar92 | 181,5 | B4 | 1 | 51 | 2 | 1.2 |
| UM | gi\|124511798 | MAL7P1.50 | 257,0 | B1 | 1 | 51 | 2 | 0.8 |
| UM | gi\|29293851 | var1 | 286,7 | C | 1 | 50 | 2 | 0.8 |
| UM | gi\|323392627 | DBLα | 215,1 | - | 1 | 50 | 2 | 13.7 |
| UM | - | PREICHvar85 | 201,8 | B4 | 1 | 49 | 2 | 1.0 |
| UM | - | HB3var06 | 399,1 | A3 | 1 | 49 | 2 | 0.6 |
| UM | gi\|124015295 | IT4var60 | 315,3 | - | 1 | 47 | 2 | 0.9 |
| UM | - | IT4var26 | 253,5 | B1 | 1 | 44 | 2 | 1.4 |
| UM | gi\|124511804 | MAL7P1.55 | 256,5 | B7 | 1 | 43 | 2 | 0.9 |
| UM | gi\|124505159 | PFD0020c | 398,2 | A1 | 1 | 43 | 2 | 0.4 |
| UM | - | HB3var35 | 248,1 | - | 1 | 39 | 2 | 0.7 |
| UM | gi\|124015303 | IT4var06 | 353,0 | B1 | 1 | 39 | 2 | 0.6 |
| UM | gi\|323393343 | DBLα | 211,0 | - | 1 | 37 | 2 | 12.0 |
| UM | - | PFCLINvar26 | 259,2 | C1 | 1 | 36 | 2 | 0.7 |
| UM | - | IGHvar24 | 323,7 | A3 | 1 | 35 | 2 | 0.9 |
| UM | gi\|90193199 | DBLα1 | 34,5 | - | 1 | 33 | 2 | 6.3 |
| UM | - | DD2var43 | 365,5 | A3 | 1 | 31 | 2 | 0.7 |
| UM | - | RAJ116var39 | 273,6 | B6 | 1 | 29 | 2 | 0.7 |
| UM | gi\|197252495 | var3 | 202,6 | B | 1 | 29 | 2 | 12.9 |
